# Senile Insanity

**Published:** 1856-02

**Authors:** John M. Galt

**Affiliations:** Superintendent and Physician of the Eastern Asylum of Virginia


*Senile Insanity.—Hypochondriasis.* By John M. Galt, M. D., Superintendent and Physician of the Eastern Asylum of Virginia.

Two individuals were, some time since, brought to the Eastern Lunatic Asylum on the same day and from the same county; they were duly committed by the magistrates of the county in question as being insane, but both of them were refused admission by the Board of Directors of the Asylum. This refusal occurred at an adjourned meeting of the Directors, they not concurring with the county authorities as to the propriety of their reception as patients. The first meeting consisted of three members, the smallest number allowed by law to adjudicate any case, and the second of seven of the eleven who constitute the Directory.

We may mention that in no state of the Union has there been a more entire immunity from any difficulties concerning unjust confinement than in Virginia, notwithstanding that her provision for the insane dates back at so remote a period, the Eastern Asylum having been founded in 1769, and having been opened for the reception of patients in the year 1773. We think this most desirable immunity greatly owing to the judicious character of the legal enactments concerning the confinement of the insane. Boarders are permitted to be sent to the asylums without the action of magistrates; but, in that event, the decision of the court of examination is required to be unanimous. In the case of state patients, equal care is found in the circumstance that the preliminary action of three magistrates is necessary, and then a subsequent examination by the Directory. It has also been a custom for each supposed lunatic to be examined by the Superintendent before coming in the presence of the Board, and a previous custom has recently been converted into a positive regulation, at least so far as concerns the co-operation of the Superintendent. Occasionally cases originally refused have been returned again, and ultimately admitted as patients; and perhaps the danger in the arrangements here adopted lies in this particular, as might be almost anticipated from the complete absence of the opposite evil; for, by the very law of compensation, evil and good are usually conjoined, to a greater or less extent, so that if you obtain an important end, it is only by some sacrifice. As to the cases discussed in the present article, the writer was doubtful what should have been the decision; and they are not given so much to show whether their rejection was erroneous or the reverse, as to form the basis for a few remarks concerning the class of individuals to which they assimilate.

As respects the general outlines of the first case, there was evidently little more, if any, mental disturbance, other than the mere weakness of mind attending old age. There was, it is true, a decided psychical debility, and especially of the memory; thus he repeated over and over again that his name was A. B., that he was cousin of C. D., that he had lost all his property by going security, &c.; moreover, he appeared unable to tell where he was, or how long he had been on the route to the Asylum. But, in relation to unmanageable conduct, there was little evidence that there had been any such; and there was none of that irritability or the like morbid state of the feelings leading to conduct quite extravagant in an old person. One of the strongest circumstances favoring the idea that insanity existed, was the fact that he had a cousin who was committed to the Asylum as insane, being so found by the jury who tried him for having killed his father in the most shocking manner, cutting him up with an axe. Under the law, he remained at the hospital until sufficiently restored, again to undergo judicial scrutiny. He died after a residence of eleven years.

In looking over the writers on the subject, we find but few observations with respect to the question of confining in asylums the sufferers from senile dementia. We are ourselves very much inclined to think that expediency should be the test here, rather than any definite line of demarcation. As there is usually but slight hope of a recovery, and the patient is not dangerous, unless under peculiar circumstances, we really consider this to be, in general, one of the instances in which relatives are bound to bear the burthen of those to whom life presents but a few years before "the golden bowl is broken," and "the wheel is broken at the cistern."

With respect to senile insanity, writers on jurisprudence have discussed largely the question of the sanity or insanity of testators suspected of this form of mental alienation. We think that remarks of this nature are usually of a not sufficiently definite character; and, doubtless, it is in practice quite difficult to say where this malady commences, and where we have to do with simple decay of the faculties, apart from any morbid change. As, however, the word, "madness"* expressed the popular view of insanity, at the same time that it was a correct term—as implying the existence of a morbid state of the feelings in lunacy, thus depicting the real features of the disease, in opposition to the false definitions of Locke and others, limiting the morbid condition to the intellect; so, in a similar mode, the distinction between mere decay of the mental powers and senile dementia, though not, as we have intimated, in such express language, yet still seems intended by writers on the subject to consist in the absence of morbid emotions on the one hand and their presence on the other. Dr. Ray has the most lucid remarks as to this topic, in his Treatise on Jurisprudence—a work that does more credit to America than aught in relation to insanity that has been produced on this side of the Atlantic. He observes: "This form of the disorder, or senile dementia, is so often the subject of medico-legal inquiries, especially in connection with wills, that it deserves particular attention. Senile dementia, it must be recollected, is something more than the mere loss of mental power which results from the natural decay of the faculties; it is not only feeble, but it is deranged. Were it not so, every old man would labor under a certain degree of dementia." Then, after alluding especially to the impairment of the memory, he says: "The first symptom indicative of derangement is a degree of incoherence in the ideas, like that of dreams, &c." Such being the ordinary course of senile dementia, he observes, in addition, that there is sometimes "great irritability, excitement of the venereal appetite,—of an appetite for high-seasoned dishes and intoxicating drinks." This last remark of Dr. Ray appears to have been borrowed from the renowned treatise of M. Esquirol, who uses almost the identical words here employed. But, as we have said, the main line of demarcation between mere natural decay of the faculties and senile dementia has never been so clearly pointed out as by Dr. Ray. Burrows, also, in his "Commentaries," observes: "In this singular affection the system is influenced by an extraordinary excitation, prompting the revival of youthful passions and follies, when the power of fruition has long ceased. The whole moral and intellectual character of the patient is changed; the pious become impious, and the content and happy discontented and miserable, the prudent and economical imprudent and ridiculously profuse, the liberal penurious, the sober drunken, &c. Persons in whom the sexual passion has been long dormant suddenly become lascivious and obscene." He mentions, as an instance, a nobleman who, up to his ninetieth year, enjoyed every faculty

*From the Gothic *mod.*

in a healthy state, and especially a clear understanding, when suddenly he became very violent and imperious in his conduct and conversation, purchased many ridiculous things, grew fond of alcoholic drinks, &c. Araeteus asserts that this mental condition is always fatal. Dr. Conolly, in his work on the "Indications of Insanity," intimates the necessary caution, that we should make a "greater degree of allowance in some cases than in others; for instance, where the individual has *always* been eccentric; for the eccentricity will probably be increased by age; and for one unacquainted with the previous habits of the patient, he may seem to be mad, although, perhaps, merely a humorist, who has in declining life become a little more childish in his humors." In the same production he mentions the singular case of an old gentleman who, on approaching his ninetieth year, experienced such an impairment of his mental faculties, that he sometimes imagined himself dead, communicating the intelligence of his own discease to his friends, with perfect gravity and an air of entire resignation, but only professing himself a little scandalized that the windows were not closed on the occasion. He would likewise direct that the sad news ought to be communicated to his friends, that he went off easily, and to conclude the matter, he requested one more pinch of snuff before he was finally screwed down in his coffin. Dr. Prichard observes, in his well-known "Treatise on Insanity:" "Senile dementia, or the decay of the mental faculties, is not the lot of old persons universally, though it is a condition to which old age may be said to have a tendency, and to which, in the last stage of bodily decay, some approximations are generally to be perceived." In his extensive work, "De la Folie, consideree dans ses rapports avec les questions medico-judiciares," strange to say, M. Marc fails to dwell on the important subject of senile insanity. There is also a deficiency here in De Boismont, Fodere, Georget, Moreau, Bottex, and other French writers. And the same may be affirmed of the leading authorities of Germany, or they fall into the opposite error of confusion. Indeed, we have been able to find no allusion to the simple distinctions of Dr. Ray and M. Esquirol, beyond an agreement with the latter. This, for example, is the case with the most elaborate Italian authors, such as Bonacossa, Fantonelli, Balletti, and other standard writers; although the first mentioned physician, besides being remarkable for his many divisions and subdivisions, has, moreover, written a great deal concerning dementia at an early period, in his inaugural thesis, and later in his very valuable work, the "Patologia Mentale."

With regard to the case of hypochondriasis alluded to in the first few words of this article, I should, perhaps, apologise for including in the same essay remarks relative to two forms of mental alienation so entirely disconnected as senile insanity and hypochondriasis. But, as has been previously explained, there was a temporary connection of circumstances leading to analogous remarks upon the two. Passing, then, to the case of hypochondriasis: in this instance the opinion of the writer was asked by the Board of Directors; and it was presented to them as a conclusion, that it was best to be guided by the wish of the sufferer himself—in other words, to leave it to him to determine whether he would become an inmate of the asylum. My reason for this suggestion, I observed, was, that, in the first place, it was true that an individual thus affected was entitled, as a right, to a position in an asylum. For, throwing aside the mere existence of delusion in hypochondriasis, we would otherwise arrive at the same opinion, because it so frequently happened that melancholia and hypochondriasis alternate, and pass into each other, that the connection is too intimate to justify us in refusing to receive such a patient. Moreover, I remarked that in both these affections, suicide is so often the lot of the unhappy victim, that a second reason is thus constituted why we should give him succor against so dire a calamity. But, on the other hand, although dangers of the kind were imminent, yet we should guard against infringing on the rights of persons, at any rate recognized by law, or entitled to the privileges of the same. For is it not notorious that individuals, with the most absurd hypochondriacal delusions, have, nevertheless, performed the duties of important civil situations, and, in addition, had the use of their property, and the power of making a will? Hence it was that I made the suggestion before mentioned to the authorities of the Eastern Asylum.

These ideas may be illustrated by reference to various writers. Thus Esquirol observes: "Hysteria and hypochondriasis often gradually become or pass into madness, and in many cases they but constitute its first stage, which, indeed, has caused many authors, both ancient and modern, to confound the two maladies." And again, in his able article on suicide, he says that "the pain which leads to lypemania and hypochondriasis often results in suicide." And the distinguished Brachet, in his extensive work, entitled "Traite Complete de L'Hypochondrie," avers that the malady most frequently terminating hypochondriasis is mental alienation. In the "Anatomy of Suicide," it is observed: "There is no more frequent cause of suicide than visceral derangement, leading to melancholia and hypochondriasis." And further, it is said: "In the case of Cowper, we have a melancholy instance of hypochondriacal feeling leading to suicidal derangement." It is from remarks of this character, in standard writers, that we are led to decide on affording him our assistance, when the victim of hypochondriasis knocks at the doors of our lunatic asylums. But, on the other hand, mere nervous feeling and hypochondriacal symptoms are too closely connected, and too often but slightly prevent the sufferer from attending to his affairs, to justify the procedure of forcibly placing an individual thus affected within the walls of an institution for the insane. In this relation, for example, Dr. Conolly, in his work on the "Indications of Insanity," warns us against acting too rigorously. Says he: "The far more important, but not more difficult duty of the practitioner, is, for the most part, neglected—that of considering, with all the caution which such a serious case requires, whether or not the departure from sound mind be of a nature to justify the confinement of the individual, and the imposition of restraint upon him, as regards the use or disposal of his property." And again, in treating of false sensations, he declares: "A man may be mad upon that point alone, and his madness may be of no consequence to himself or others." Additional weight, too, must be assigned to considerations of the sort, when we turn our attention, not only to ordinary cases, but to certain instances well known to the literary public. Thus, Boswell, after stating that Dr. Johnson was a hypochondriac, goes on to say: "Though he suffered severely from it, he was not therefore degraded. The powers of his great mind might be troubled, and their full exercise suspended at times; but the mind itself was ever entire. As a proof of this, it is only necessary to consider, that, when he was at the very worst, he composed that state of his own case, which showed an uncommon vigor, not only of fancy and taste, but of judgment." In Cowper, the hypochondriacal element, taking the form of an excessive dread of punishment in the next world, ended in positive insanity of a suicidal character; whilst in Dr. Johhson, the morbid predisposition stopped short of this, though he too appears to have experienced an inordinate apprehension of death and hereafter.

Many passages may be found in writers on insanity touching those degrees of mental disturbance not evincing the broad and unmistakable features of positive and decided lunacy. Dr. Conolly observes, for example: "So many hypochondriacal persons are known to be at large who entertain strange opinions concerning their own form and nature, that it seems hardly necessary to caution the practitioner against treating such patients as madmen are commonly treated." And elsewhere he says: "Harrington, the author of the 'Oceana,' cherished a notion that his animal spirits transpired from him in the shape of birds, or flies, or bees; much of his conversation turned on good and evil spirits; and he would use strong arguments to prove that his sensorial illusions were realities: but on other subjects he was clear and rational."

I could also particularize cases, reported to me by reliable persons, of individuals of their acquaintance laboring under a similar mental disturbance—a disturbance where no compulsory measures would have been deemed at all justifiable. Thus a lady, in one instance, was almost deprived of the pleasure of a drive, because, if she rode in town, she complained of being constantly apprehensive lest the houses should fall and crush her, and if in the country, that an analogous calamity would ensue from falling trees. A second was always strongly affected by the sight of her housekeeper when in a particular dress, insomuch that, on once returning home and meeting her so clad at the porch, she fainted away. She also occasionally fancied herself a tea-urn, and, under that impression, would assume a suitably imitative position. This was likewise the case with a gentleman mentioned to me, and with a third lady. The last occasionally imagined, besides, that she was a goose, and, in accordance with the supposition, would sometimes be found crouched upon a parcel of carpet-rags in a little closet, and, when approached, would hiss very anserously, so as to fully entitle herself, in the opinion of the ill-natured, to an appellation corresponding with the character which she had assumed; yet, if her friends called to pay her a visit, she would desist from her absurd conduct, and talk to and entertain them rationally during the whole evening. Under the present head may be included, also, the strange antipathies reported by writers In this class I have heard of three individuals whose nerves would be extremely affected, merely by the entrance of a cat into the room where they were.—*Am. Jour. of Insanity.*

